# Ambient Fine Particulate Matter and Mortality among Survivors of Myocardial Infarction: Population-Based Cohort Study

**DOI:** 10.1289/EHP185

**Published:** 2016-05-06

**Authors:** Hong Chen, Richard T. Burnett, Ray Copes, Jeffrey C. Kwong, Paul J. Villeneuve, Mark S. Goldberg, Robert D. Brook, Aaron van Donkelaar, Michael Jerrett, Randall V. Martin, Jeffrey R. Brook, Alexander Kopp, Jack V. Tu

**Affiliations:** 1 Public Health Ontario, Toronto, Ontario, Canada; 2 Dalla Lana School of Public Health, University of Toronto, Toronto, Ontario, Canada; 3 Institute for Clinical Evaluative Sciences, Toronto, Ontario, Canada; 4 Population Studies Division, Health Canada, Ottawa, Ontario, Canada; 5 Department of Family and Community Medicine, University of Toronto, Toronto, Ontario, Canada; 6 Department of Health Sciences, Carleton University, Ottawa, Ontario, Canada; 7 Department of Medicine, McGill University, Montreal, Quebec, Canada; 8 Division of Clinical Epidemiology, McGill University Health Centre, Montreal, Quebec, Canada; 9 Division of Cardiovascular Medicine, University of Michigan Medical School, Ann Arbor, Michigan, USA; 10 Department of Physics and Atmospheric Science, Dalhousie University, Halifax, Nova Scotia, Canada; 11 Division of Environmental Health Sciences, School of Public Health, University of California, Berkeley, Berkeley, California, USA; 12 Harvard-Smithsonian Center for Astrophysics, Cambridge, Massachusetts, USA; 13 Air Quality Research Division, Environment Canada, Toronto, Ontario, Canada; 14 Division of Cardiology, Schulich Heart Centre, Sunnybrook Health Sciences Centre, Toronto, Ontario, Canada

## Abstract

**Background:**

Survivors of acute myocardial infarction (AMI) are at increased risk of dying within several hours to days following exposure to elevated levels of ambient air pollution. Little is known, however, about the influence of long-term (months to years) air pollution exposure on survival after AMI.

**Objective:**

We conducted a population-based cohort study to determine the impact of long-term exposure to fine particulate matter ≤ 2.5 μm in diameter (PM2.5) on post-AMI survival.

**Methods:**

We assembled a cohort of 8,873 AMI patients who were admitted to 1 of 86 hospital corporations across Ontario, Canada in 1999–2001. Mortality follow-up for this cohort extended through 2011. Cumulative time-weighted exposures to PM2.5 were derived from satellite observations based on participants’ annual residences during follow-up. We used standard and multilevel spatial random-effects Cox proportional hazards models and adjusted for potential confounders.

**Results:**

Between 1999 and 2011, we identified 4,016 nonaccidental deaths, of which 2,147 were from any cardiovascular disease, 1,650 from ischemic heart disease, and 675 from AMI. For each 10-μg/m3 increase in PM2.5, the adjusted hazard ratio (HR10) of nonaccidental mortality was 1.22 [95% confidence interval (CI): 1.03, 1.45]. The association with PM2.5 was robust to sensitivity analyses and appeared stronger for cardiovascular-related mortality: ischemic heart (HR10 = 1.43; 95% CI: 1.12, 1.83) and AMI (HR10 = 1.64; 95% CI: 1.13, 2.40). We estimated that 12.4% of nonaccidental deaths (or 497 deaths) could have been averted if the lowest measured concentration in an urban area (4 μg/m3) had been achieved at all locations over the course of the study.

**Conclusions:**

Long-term air pollution exposure adversely affects the survival of AMI patients.

**Citation:**

Chen H, Burnett RT, Copes R, Kwong JC, Villeneuve PJ, Goldberg MS, Brook RD, van Donkelaar A, Jerrett M, Martin RV, Brook JR, Kopp A, Tu JV. 2016. Ambient fine particulate matter and mortality among survivors of myocardial infarction: population-based cohort study. Environ Health Perspect 124:1421–1428; http://dx.doi.org/10.1289/EHP185

## Introduction

Acute myocardial infarction (AMI) is one of the most common cardiovascular events, affecting ~7.9 million adults in the United States (Roger et al. 2011) and 540,000 in Canada (Chow et al. 2005).

Once people develop an AMI, their chances of long-term survival and their quality of life are reduced substantially (Roger et al. 2011). Recent studies have shown that people with an AMI had induced ST segment depression (Mills et al. 2007), decreased heart-rate variability (Park et al. 2005; Zanobetti et al. 2010), and increased ischemic events (Pope et al. 2006) within several days after exposure to elevated levels of air pollution. People with an AMI have also been found to be at higher risk of dying when daily pollution levels increase, particularly with particulate matter ≤ 10 μm in diameter (PM_10_) (Bateson and Schwartz 2004; Berglind et al. 2009; von Klot et al. 2005). These findings are supported by toxicological studies linking pollution with increased systemic oxidative stress and inflammation, blood coagulability, progression of atherosclerosis, and reduced heart-rate variability (Brook et al. 2010), indicating that AMI patients may be particularly sensitive to air pollution exposure (O’Neill et al. 2012; Sacks et al. 2011).

Little is known, however, about the influence of long-term (months to years) exposure to air pollution on mortality after AMI, although there is increasing evidence that long-term exposures result in substantially larger health risks than exposures over several days (Brook et al. 2010). Among a small set of studies that have assessed the influence of long-term exposure to air pollution on mortality after AMI, three studies reported increased all-cause mortality in association with exposure to PM_2.5_ (particles ≤ 2.5 μm in diameter) (Tonne and Wilkinson 2013), PM_10_ (Zanobetti and Schwartz 2007), and elemental carbon (C) (von Klot et al. 2009). However, in two other studies, no compelling evidence was found for associations with PM_2.5_ (Koton et al. 2013) or nitrogen dioxide (NO_2_) (Rosenlund et al. 2008). Because cause-of-death information was unavailable in previous studies (Tonne and Wilkinson 2013; von Klot et al. 2009; Zanobetti and Schwartz 2007), the specificity of the association between air pollution and post-AMI mortality remains uncertain; understanding this association would be helpful for elucidating pathways linking long-term exposure with survival in this subpopulation.

Therefore, we conducted a population-based cohort study to evaluate the impact of long-term exposure to PM_2.5_ on survival among AMI patients. In addition, we sought to quantify the burden of post-AMI mortality attributed to PM_2.5_. Given the high prevalence of AMI and the ubiquity of air pollution, such information may help target interventions to improve outcomes for AMI patients.

## Methods

### Study Design and Study Population

We conducted a cohort study of newly admitted AMI patients participating in Phase 1 of the Enhanced Feedback For Effective Cardiac Treatment (EFFECT) study (1999–2001) (Tu et al. 2009), a large randomized trial in Ontario, Canada. Details of the EFFECT study have been presented elsewhere (Tu et al. 2009). Briefly, that study included all patients admitted to one of 86 hospital corporations in Ontario with a primary or most responsible diagnosis of AMI (*International Classification of Diseases, Ninth Revision*, ICD-9 code 410). Trained nurses abstracted demographic (e.g., marital status) and clinical (e.g., smoking status, laboratory tests, and medical history) information from patients’ primary charts. After we reviewed the medical records, patients who *a*) fulfilled the European Society of Cardiology/American College of Cardiology clinical criteria (Alpert et al. 2000), *b*) had AMI onset before arriving at the hospital, and *c*) were registered with Ontario’s provincial health insurance plan were included (Tu et al. 2009). Patients transferred from other acute-care facilities were excluded.

We restricted the study population to those who were ≥ 35 years of age at hospital admission, had a length of hospital stay of ≥ 2 days, and were Canadian-born individuals. Consistent with previous studies of air pollution and post-AMI survival (Berglind et al. 2009; Rosenlund et al. 2008; Tonne and Wilkinson 2013; von Klot et al. 2005), we further restricted the study population to those who were alive for ≥ 28 days after hospital discharge.

The Research Ethics Board of Sunnybrook Health Sciences Center, Toronto, approved the study.

### Outcomes

The follow-up period was from the 29th day after discharge in 1999–2001 until the end of 2011. We ascertained the underlying cause of death and the date of death using record linkage to the Ontario Registrar General’s Death database using the patient's unique, encrypted health card number (linkage rate: 98%). The primary outcome was nonaccidental mortality (ICD-9 codes are listed in Table S1). To evaluate the specificity of the association between air pollution and mortality, we also ascertained deaths from any cardiovascular disease, ischemic heart disease, and AMI. In addition, to detect possible bias because of unmeasured confounding and other errors that may lead to spurious inference, we considered negative control outcomes for which no (or weaker) associations with air pollution were expected (Lipsitch et al. 2010). To do this, we identified deaths from accidental causes and from noncardiopulmonary, non–lung cancer causes (Jerrett et al. 2013).

### Assessment of Ambient Concentrations of PM_2.5_

**Figure 1:**
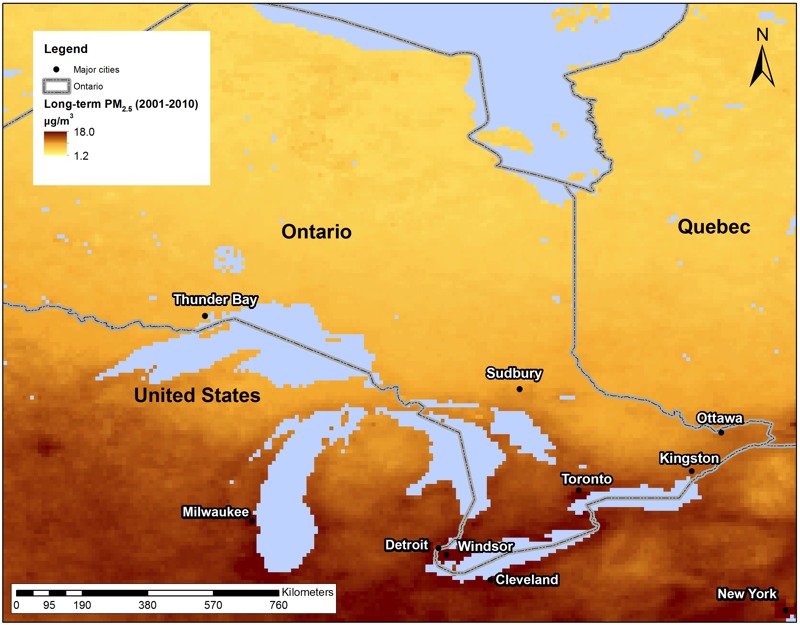
Mean satellite-derived estimates of PM_2.5_ across Ontario, Canada, 2001–2010.

Estimates of ground-level concentrations of PM_2.5_ were derived from satellite observations of aerosol optical depth [sources of AOD are publicly available and were downloaded from ftp://ladsweb.nascom.nasa.gov (MODIS Terra and Aqua) and ftp://l4ftl01.larc.nasa.gov (MISR); the data were obtained over several years up to 2013, and version control maintained consistency throughout the access period], a measure of light extinction by aerosols in the total atmospheric column, in conjunction with outputs from a global atmospheric chemistry transport model (GEOS-Chem CTM) (van Donkelaar et al. 2015). We used estimates from 2001 to 2010, thus obtaining 10-year mean concentrations of PM_2.5_ at a resolution of approximately 10 km × 10 km and covering North America below 70°N, which includes all of Ontario (Figure 1). These satellite-based estimates of PM_2.5_ are in good accord with ground measurements at fixed-site stations across North America (Pearson correlation coefficient *r* = 0.76, *n* = 974) (van Donkelaar et al. 2015), and they improve the accuracy and spatiotemporal coverage of our earlier satellite-based estimates of PM_2.5_ (van Donkelaar et al. 2010), which have been used to determine the associations of PM_2.5_ with mortality and morbidity (Chen et al. 2013; Crouse et al. 2012), as well as the global disease burden attributable to air pollution (Lim et al. 2012).

The location of residence for each participant during the follow-up period was obtained from the Registered Persons Database, a registry of all Ontario residents with health insurance (Chen et al. 2013). Locations were refined to the spatial scale provided by six-character postal codes, which in urban areas represent a city block or a large apartment complex. We created annual estimates of exposure to PM_2.5_ for each participant by interpolating the 10-year mean concentrations of PM_2.5_ to the centroid of their residential postal code for that year, thereby accounting for residential mobility. This approach assumed that the spatial pattern in PM_2.5_ did not change appreciably during follow-up (Miller et al. 2007; Pope et al. 2002). This assumption is reasonable because we have shown previously that areas in Ontario with high concentrations of PM_2.5_ have retained their spatial ranking from 1996 to 2010 and that variability in long-term exposure to PM_2.5_ is primarily spatial rather than temporal (Chen et al. 2013).

### Covariates

We selected *a priori* the following potential confounders, abstracted from medical records: age, sex, marital status, employment status (employed/unemployed/retired/homemaker/disabled), major cardiac risk factors [including smoking status, family history of coronary artery disease, diabetes, hyperlipidemia, hypertension, stroke, previous AMI, and previous percutaneous coronary intervention (PCI)], AMI type [ST elevation/non-ST elevation (STEMI/non-STEMI)], acute pulmonary edema, selected comorbidities (including angina, cancer, dementia, dialysis, and chronic obstructive pulmonary disease), and cardiovascular medications at hospital discharge [including statins, aspirin, angiotensin converting enzyme (ACE) inhibitors, and beta-blockers]. To assess in-hospital care, we obtained information about the length of hospital stay (days) and the characteristics of attending physicians (cardiologist/internist/family physician) and hospitals (teaching/community/small) (Tu et al. 2009). In addition, to assess the severity of the AMI, we calculated the Global Registry of Acute Cardiac Events (GRACE) risk score based on age, history of congestive heart failure and AMI, heart rate, systolic blood pressure, and several other prognostic variables (Bradshaw et al. 2006). We also derived body mass index (BMI; kg/m^2^) using self-reported height and weight.

Using 2001 Canadian census-tract data (see Supplemental Material, “Canadian Census Divisions and Census Tracts”), we derived three neighborhood-level variables: *a*) percentage of population ≥ 15 years of age with less than high school education; *b*) unemployment rate; and *c*) mean household income. To control for region-scale spatial patterns in mortality that might be caused by factors other than pollution, we created a dichotomous variable classifying Ontario into the Greater Toronto area, a densely-populated urban megaregion, and all other areas. Toronto tends to differ from other areas in Ontario with respect to socioeconomic and demographic characteristics, health care, and mortality rates (see Table S2).

### Statistical Analysis

Standard and multilevel spatial random-effects Cox proportional hazards models (Ma et al. 2003) were used to assess post-AMI mortality in relation to PM_2.5_. The spatial random-effects model accounted for the possibility that patterns of health of residents living in the same or neighboring communities were more similar than for individuals living further apart and that these patterns may not be completely explained by variables included in the model. This modeling approach has been used extensively in previous studies of pollution-related mortality in the United States (Jerrett et al. 2013; Pope et al. 2002, 2004) and in Canada (Crouse et al. 2012).

Consistent with previous studies (Crouse et al. 2012), the random effects in our spatial random-effects Cox model were represented by two levels of spatial clusters, with a first cluster level defined by census divisions (equivalent to counties) and a second level defined by census tracts within census divisions. We assumed that two census divisions were correlated if they were adjacent, and we made the same assumption for adjacent census tracts within each census division. Census tracts in different census divisions were assumed to be uncorrelated.

We developed models for mortality from nonaccidental causes, cardiovascular (any, ischemic heart, AMI), and as negative controls, accidental and noncardiopulmonary, non–lung cancer causes. We stratified the baseline hazard function by single-year age groups and by region, allowing each category to have its own baseline hazard. We included participants with nonmissing information on exposure and covariates, except for marital status (~3% of the cohort had unknown values), employment status (6%), smoking (12%), and BMI (41%), for which we created a separate category of missing values to avoid losing substantial statistical power.

We measured follow-up time (in days) from baseline until death (47%), ineligibility for provincial health insurance (2%), or end of follow-up (51%). We fitted PM_2.5_ as a time-varying variable by modeling time-weighted exposure from baseline until death, with weights for each individual defined by the time spent at each residence. We constructed a sequence of models including different potential confounding factors (see Figure S1). The final model included variables for sex, marital status, employment status, smoking status, family history of coronary artery disease, diabetes, hyperlipidemia, hypertension, stroke, previous PCI, previous AMI, GRACE risk score, AMI type, acute pulmonary edema, indicators for in-hospital care, medications at discharge, comorbidities, and ecological variables. We adjusted for regional variations in the ecological variables across Ontario using the average for each census division and the difference between the values for each census tract and the census division mean. Because of the considerable missing data for BMI (41%), we did not include it in the main model, but we considered it in a sensitivity analysis.

**Figure 2:**
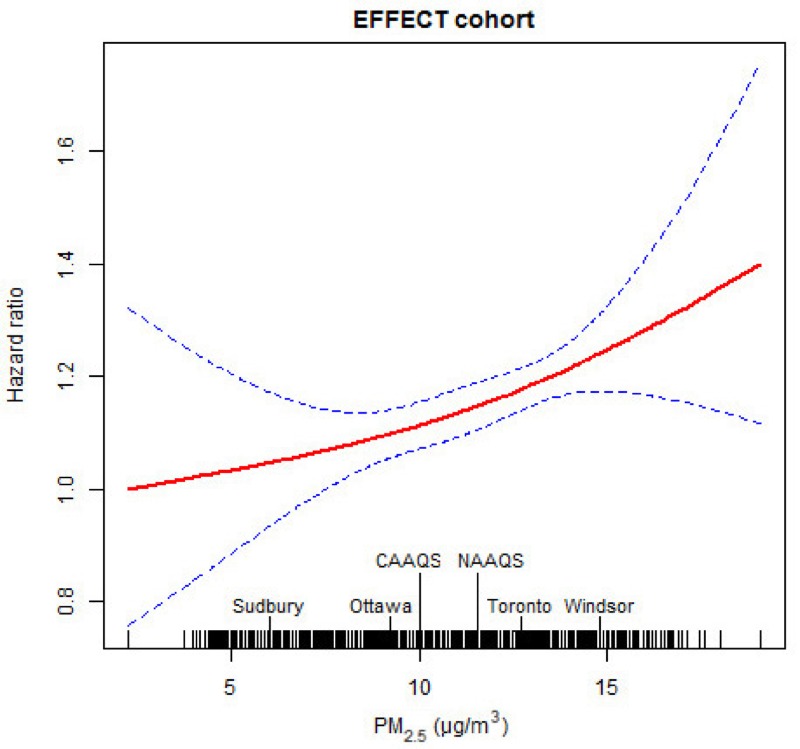
Concentration–response relationship between the concentration of particles with diameter ≤ 2.5 μm (PM_2.5_) and nonaccidental mortality during 13-year follow-up after acute myocardial infarction. The hazard ratios were estimated by comparing with 2.2 μg/m^3^. The city-mean concentrations of PM_2.5_ for four selected cities in Ontario, the current Canadian Ambient Air Quality Standards (CAAQS, objectives for annual mean concentration: 10 μg/m^3^), and the U.S. National Ambient Air Quality Standards (NAAQS, standards for annual mean concentration: 12 μg/m^3^) for PM_2.5_ are indicated.

We tested for deviations from the proportional hazards assumption by adding the cross product of each variable to the natural logarithm of the time variable, but we did not find any violations of this assumption (*p* > 0.05). We also verified the assumption of linearity for all continuous variables by using natural cubic spline functions with ≤ 4 degrees of freedom (df). We examined plots of concentration–response curves for PM_2.5_ and computed the Akaike Information Criteria (AIC) to determine whether the response function was nonlinear. Because there was no evidence of departure from linearity for the relationship between PM_2.5_ and mortality (see Figure 2, see also Table S3), we report adjusted hazard ratios (HRs) and 95% confidence intervals (CIs) for each 10 μg/m^3^ increase of PM_2.5_ (referred to as HR_10_).

### Sensitivity Analyses

We performed a series of sensitivity analyses by considering follow-up starting 1 year after discharge, controlling for BMI in a subcohort with complete information, restricting the analysis to those living outside Toronto, and controlling for population density at the census-division level. In addition, we further controlled for distance to nearest acute-care hospital using a natural cubic spline with 3 df, adjusted for coronary revascularization during follow-up as a time-varying variable, and adjusted for a categorical variable indicating the population size of participants’ home communities (rural, < 30,000, 30,000–99,999, 100,000–499,999, ≥ 500,000). We obtained information about coronary revascularization through data linkage to the Discharge Abstract Database and the Ontario Health Insurance Plan Claims Database (Chen et al. 2013).

In addition, to account for time trends in air pollution and mortality, we also controlled for calendar time using a natural cubic spline with 3 df. To investigate whether the hazard ratios might be influenced by any possible spatial dependence introduced by hospitals, we further added a frailty term (random effect) for hospitals to allow for the possibility that the effect estimates varied from hospital to hospital in the estimation of the main effect and its variance. A gamma distribution for random effect was assumed, with an exchangeable correlation structure within hospitals. We compared the models with and without a frailty term using the AIC. Furthermore, we additionally controlled for neighborhood-level deprivation, density of family physicians, and several other geographically variable sociodemographic and health care indicators; we also restricted to cohort members living between 41.7°N and 46.0°N, where the vast majority of the Ontario population resides, and to those living within 5 km of any manufacturing or process facilities releasing particulate matter (see Supplemental Material, “Additional Sensitivity Analyses”).

Lastly, we investigated *a priori* whether individuals with preexisting angina, AMI, diabetes, and hypertension were at greater risk, as well as testing for potential effect modification by AMI type (STEMI/non-STEMI) and medication use, by assessing whether the interaction term that was the cross-product of each variable with the PM_2.5_ value was statistically significant.

### Burden Attributable to PM_2.5_

To quantify the burden of death attributed to long-term exposure to PM_2.5_ among those with AMI, we estimated the number of deaths caused by PM_2.5_ with reference to an alternative (counterfactual) distribution of exposure (i.e., the minimum that could be achieved at the population level). For this procedure, we chose the lowest concentration of PM_2.5_ measured across all cities worldwide (4 μg/m^3^) (Brauer et al. 2012). We then derived the attributable fraction and applied it to the number of nonaccidental deaths during follow-up (see Supplemental Material, “Burden Attributable to PM_2.5_”). We used the hazard ratio from the fully adjusted spatial random-effects model. To estimate the 95% uncertainty interval, we sampled the 2.5th and 97.5th percentile of 1,000 draws from the distribution of exposure and the hazard ratio using the approach reported in the Global Burden of Disease Study 2010 (Lim et al. 2012).

Analyses were performed using the R statistical package (v.3.0.0, 64-bit) (R Core Team 2015). The spatial random-effects Cox model was fitted using the Cox-Poisson program (Krewski et al. 2009).

## Results

Among the 10,386 eligible patients from the EFFECT study, we excluded 84 (~1%) patients who were < 35 years old, 379 (4%) whose length of hospital stay was < 2 days, 281 (3%) who were landed immigrants, 284 (3%) who died within 28 days post-discharge, and 485 (5%) with missing data on covariates except for marital status, employment status, smoking, and BMI, leaving a total of 8,873 patients in our analytical cohort.

At the time of entry, the mean age was 66.9 years, 65% were men, and 36% were current smokers (Table 1). Of the cohort, 23% had a prior AMI, nearly half had been diagnosed with STEMI, and 34.5% were prescribed statins at discharge. Average unemployment among the census tracts was 6%, and the mean household income was 52,400 CAD.

**Table 1:** Baseline characteristics of the study population.

Baseline characteristics^*a*^	Cohort (*n* = 8,873)
Demographic characteristics
Age, years	66.9 ± 13.0
Men, %	65
Marital status, %
Married	68
Single	6
Separated, widowed, or divorced	23
Unknown	3
Employment, %
Employed/self	26
Homemaker	3
Retired	62
Unemployed	1
Disabled	2
Unknown	6
Cardiac risk factors and history
Smoking, %
Never smoker	28
Current smoker	36
Former smoker	24
Unknown	12
Body mass index, kg/m^2^	27.9 ± 5.5
< 18.5 (%)	1
18.5–24.9 (%)	17
25.0–29.9 (%)	25
≥ 30.0 (%)	16
Unknown (%)	41
Family history of coronary artery disease, %	33
Diabetes, %	25
Hyperlipidemia, %	32
Hypertension, %	46
Previous percutaneous coronary intervention, %	3
Previous myocardial infarction, %	23
Stroke, %	7
GRACE risk score	142 ± 36
Comorbidities, %
Angina	32
Cancer	3
Dementia	3
Dialysis	1
Chronic obstructive pulmonary disease	1
Clinical risk parameters, %
ST elevation myocardial infarction	49
Acute pulmonary edema	5
In-hospital care
Length of stay, days	8.0 ± 7.8
Specialty of attending physician, %
General practice	34
Internal medicine	31
Cardiology	35
Characteristics of hospitals, %
Teaching	13
Community	80
Small	7
Cardiovascular medication at discharge, %
Statins	35
Aspirin	78
ACE inhibitor	55
Beta-blockers	70
Area-level characteristics^*b*^
Percentage population aged ≥ 15 years with less than a high school education	29
Percentage population aged ≥ 15 years without employment	6
Average household income (1,000 CAD)	52.4 ± 23.7
Abbreviations: ACE, angiotensin-converting enzyme; GRACE, Global Registry of Acute Coronary Events. ^***a***^Values are percent or mean ± standard deviation. ^***b***^At the Canadian census-tract level.	

The cohort contributed 72,101 person-years of observation, with a mean follow-up of 8.1 years. During the follow-up period, ~39% of participants changed addresses, and 22% moved out of the city that they had lived in when they enrolled. The average concentration of PM_2.5_ according to participants’ residences at baseline was 10.7 μg/m^3^ (range, 2.2–16.5), with the highest average concentrations in southern Ontario (Figure 1). Between 1999 and 2011, we identified 4,016 deaths from nonaccidental causes, of which 2,147 were from any cardiovascular disease, 1,650 were from ischemic heart disease, and 675 were from AMI. In addition, there were 121 deaths caused by accidents and 1,382 deaths from noncardiopulmonary, non–lung cancer causes.

We found a positive association for nonaccidental mortality using the standard Cox model, with a hazard ratio of 1.12 (95% CI: 0.98, 1.29) with each 10-μg/m^3^ increase in PM_2.5_, after adjusting for age and sex (Table 2). The corresponding HR_10_ from the random-effects model was 1.14 (95% CI: 0.99, 1.32). Controlling for smoking, diabetes, AMI type, GRACE risk score, medication use, and several other individual-level factors strengthened the association in both models (HR_10_ = 1.18 for the standard Cox model and HR_10_ = 1.20 for the random-effects model). In models adjusting for all individual- and neighborhood-level covariates, the HR_10_ from the standard Cox model was 1.21 (95% CI: 1.03, 1.41), and the HR_10_ from the random-effects model was 1.22 (95% CI: 1.03, 1.45). Because the estimates were similar for the two models, only results from the random-effects model are reported below because this model allowed for more accurate estimation of statistical error.

**Table 2:** Association of non-accidental mortality with every 10-μg/m^3^ increase in PM_2.5_.

Model	Standard Cox model	Random-effects model^*a*^
	Hazard ratio (95% CI)	Hazard ratio (95% CI)
PM_2.5_ adjusted for sex and stratified by age and region^*b*^	1.12 (0.98, 1.29)	1.14 (0.99, 1.32)
+ Marital status, employment^*c*^	1.14 (1.00, 1.30)	1.15 (1.00, 1.33)
+ Cardiac risk factors and history^*d*^	1.16 (1.01, 1.33)	1.16 (0.99, 1.36)
+ Clinical severity parameters^*e*^	1.14 (0.99, 1.32)	1.14 (0.97, 1.34)
+ Length of stay, characteristics of physicians and hospitals	1.21 (1.05, 1.40)	1.22 (1.04, 1.43)
+ Medication use at hospital discharge^*f*^	1.20 (1.03, 1.39)	1.21 (1.03, 1.43)
+ Preexisting angina, cancer, dementia, COPD, dialysis	1.18 (1.02, 1.36)	1.20 (1.02, 1.41)
+ Area-level variables^*g*^	1.21 (1.03, 1.41)	1.22 (1.03, 1.45)
Abbreviations: CI, confidence interval; COPD; chronic obstructive pulmonary disease; PM_2.5_, particles ≤ 2.5 μm in diameter. ^***a***^A nested, spatial random-effects Cox model comprising two levels of spatial clusters: a first cluster level defined by census divisions and a second level by census tracts. ^***b***^Region: living or not in the Greater Toronto Area. ^***c***^Variables were added to the model including base model and all previous variables labeled with “+”. ^***d***^Included smoking, family history of coronary artery disease, diabetes, hyperlipidemia, hypertension, stroke, previous percutaneous coronary intervention (PCI), acute myocardial infarction (AMI), and Global Registry of Acute Coronary Events (GRACE) risk score. ^***e***^Included ST elevation myocardial infarction (STEMI)/non-STEMI and acute pulmonary edema. ^***f***^Included statins, aspirin, angiotensin-converting enzyme (ACE) inhibitors, and beta-blockers. ^***g***^Included census-division level unemployment rate, education, and annual household income, as well as the subtraction of these variables at the census-tract level from their census-division mean.		

In sensitivity analyses, the HR_10_ estimates were not appreciably altered after considering follow-up starting 1 year after discharge, adjusting for distance to nearest hospitals, adjusting for coronary revascularization during follow-up, adding a frailty term for hospitals to allow for potential spatial clustering, or other sensitivity analyses, with the exception of controlling for BMI (Table 3, see also Table S4). We found a stronger association for nonaccidental mortality in the subcohort with information on BMI (HR_10_ = 1.46, 95% CI: 1.18, 1.81), after further controlling for BMI.

**Table 3:** Sensitivity analyses for the association of nonaccidental mortality with every 10-μg/m^3^ increase in PM_2.5_.

Sensitivity analysis	Number of deaths	Nonaccidental mortality^*a*^
		Hazard ratio (95% CI)
Follow-up starting 1 year after discharge	3,301	1.19 (0.99, 1.40)
Restricted to participants with complete data on BMI	2,213	1.46 (1.18, 1.81)
Restricted to participants outside Toronto	3,046	1.28 (1.06, 1.58)
Adjusted for population density^*b*^	4,016	1.30 (1.07, 1.58)
Adjusted for distance to nearest acute-care hospital	4,016	1.22 (1.03, 1.46)
Adjusted for coronary revascularization during follow-up	4,016	1.22 (1.02, 1.44)
Adjusted for long-term time trend in calendar year	4,016	1.23 (1.03, 1.46)
Adjusted for indicators for urban size^*c*^	4,016	1.28 (1.06, 1.55)
Added a random effect for hospitals to further investigate spatial dependency as a source of bias	4,016	1.21 (1.01, 1.46)
Abbreviations: BMI, body mass index; CI, confidence interval, PM_2.5_; particles ≤ 2.5 μm in diameter. ^***a***^A nested, spatial random-effects Cox model, stratified by age and region, and adjusted for sex, marital status, employment, smoking, family history of coronary artery disease, diabetes, hyperlipidemia, hypertension, stroke, previous percutaneous coronary intervention (PCI), acute myocardial infarction (AMI), Global Registry of Acute Coronary Events (GRACE) risk score, ST elevation myocardial infarction (STEMI)/Non-STEMI, acute pulmonary edema, in-hospital care, medications, comorbidities, and area-level variables. ^***b***^At the Canadian census division level. ^***c***^Size of subjects’ home community: rural/farm; small town (< 30,000); Urban 3 (30,000–99,999); Urban 2 (100,000–499,999); and Urban 1 (> 499,999).		

We also observed stronger associations between PM_2.5_ exposure and mortality from cardiovascular disease (HR_10_ = 1.35, 95% CI: 1.09, 1.67), mortality from ischemic heart disease (HR_10_ = 1.43, 95% CI: 1.12, 1.83), and mortality from AMI (HR_10_ = 1.64, 95% CI: 1.13, 2.40) (Table 4). No association was found for mortality from accidental and noncardiopulmonary non–lung cancer causes. Furthermore, an analysis of selected subgroups did not provide compelling evidence supporting effect modification of PM_2.5_ by diabetes (*p*-interactions varied from 0.06 to 0.90 depending on the outcomes), AMI type (*p*-interactions: 0.07 to 0.33), statins (*p*-interactions: 0.43 to 0.98), and other selected characteristics.

**Table 4:** Associations of cause-specific mortality with every 10-μg/m^3^ increase in PM_2.5_.

Cause of death	ICD-9 code	Number of deaths	Fully adjusted model^*a*^
			Hazard ratio (95% CI)
Any cardiovascular	401–459	2,147	1.35 (1.09, 1.67)
Ischemic heart	410–414	1,650	1.43 (1.12, 1.83)
Myocardial infarction	410	675	1.64 (1.13, 2.40)
Non-cardiopulmonary, non-lung cancer	< 401, 520–799, and not 162	1,382	1.06 (0.81, 1.39)
Accidental	≥ 800	121	1.07 (0.41, 2.76)
Abbreviations: CI, confidence interval; ICD-9, *International Classification of Diseases*, Revision 9; PM_2.5_, particles ≤ 2.5 μm in diameter. ^***a***^A nested, spatial random-effects Cox model, stratified by age and region, and adjusted for sex, marital status, employment, smoking, family history of coronary artery disease, diabetes, hyperlipidemia, hypertension, stroke, previous percutaneous coronary intervention (PCI), acute myocardial infarction (AMI), Global Registry of Acute Coronary Events (GRACE) risk score, ST elevation myocardial infarction (STEMI)/Non-STEMI, acute pulmonary edema, in-hospital care, medications, comorbidities, and area-level variables.			

Lastly, we calculated that the rate of mortality would be reduced by 12.4% (95% CI: 1.6%, 22.5%) if this cohort had been exposed to the lowest measured level of PM_2.5_ in an urban area as opposed to their present distribution of exposure. This estimate translates to 497 (95% CI: 65, 904) deaths attributable to elevated PM_2.5_ exposure in this cohort.

## Discussion

In this cohort study of AMI patients, exposure to ambient PM_2.5_ was associated with increased nonaccidental mortality, with HR_10_ varying between 1.21 (95% CI: 1.03, 1.41) and 1.22 (95% CI: 1.03, 1.45) depending on model structures. The association was robust to sensitivity analyses and appeared to be stronger for mortality from cardiovascular causes, particularly from ischemic heart disease (HR_10_ = 1.43) and AMI (HR_10_ = 1.64). Additionally, we did not find strong evidence for effect modification by selected characteristics such as comorbidities and secondary prevention measures. Overall, our estimated association of PM_2.5_ and mortality translates to 497 deaths in this cohort (or 12.4% of nonaccidental deaths) that could have been averted if the lowest measured PM_2.5_ concentration in an urban area (4 μg/m^3^) had been achieved over the course of the study.

Few studies have investigated the relationship between post-AMI mortality and long-term air pollution exposure. In a cohort study of 154,204 AMI survivors in England and Wales with follow-up from 2004 to 2010, Tonne and Wilkinson (2013) reported an adjusted HR_10_ of all-cause mortality with PM_2.5_ of 1.20 (95% CI: 1.04, 1.38) and an HR of 1.01 (95% CI: 0.98, 1.04) per 10 μg/m^3^ of NO_2_. A second study of 1,120 AMI survivors in central Israel reported a positive but statistically nonsignificant association of PM_2.5_ with post-AMI mortality (HR_10_ = 1.3, 95% CI = 0.8, 2.1) (Koton et al. 2013). Similarly, two separate cohort studies in the United States linked increased all-cause deaths among AMI patients to PM_10_ (Zanobetti and Schwartz 2007) and elemental carbon (a proxy for traffic particles) (von Klot et al. 2009). In contrast, no association was found for NO_2_ with post-AMI survival in an Italian cohort of AMI patients (Rosenlund et al. 2008).

Our risk estimates for PM_2.5_ and mortality appeared higher than those reported previously from cohort studies based on general populations (Cesaroni et al. 2013; Crouse et al. 2012; Hoek et al. 2013; Jerrett et al. 2013; Laden et al. 2006; Pope et al. 2004). In a Canadian national cohort study following 2.1 million adults over 1991–2001, Crouse et al. (2012) reported positive associations of PM_2.5_ and mortality from nonaccidental causes (HR_10_ = 1.15), any cardiovascular disease (HR_10_ = 1.16), and ischemic heart disease (HR_10_ = 1.31). A meta-analysis of 11 cohort studies examining air pollution and cardiovascular-related mortality reported a pooled HR_10_ of 1.11 (95% CI: 1.05, 1.16) for PM_2.5_ (Hoek et al. 2013). Although there is some overlap in estimates of risk between the present study and these previous studies (Cesaroni et al. 2013; Crouse et al. 2012; Hoek et al. 2013; Laden et al. 2006; Pope et al. 2004), the higher risk estimates observed in the present cohort, particularly for cardiovascular-related mortality, suggest that AMI survivors are more susceptible to air pollution than the general population. It is noteworthy that ambient concentrations of PM_2.5_ in Ontario (annual mean in 2000: 11.2 μg/m^3^) were considerably lower than those observed in many cities in the United States (e.g., annual mean PM_2.5_ in Los Angeles: 20.7 μg/m^3^ in 2000) (Coogan et al. 2012), in Europe (e.g., Rome, Italy: 19.9 μg/m^3^ in 2010) (Cesaroni et al. 2013), and in Asia (e.g., Beijing, China: 56.0 μg/m^3^ in 2010; Delhi, India: 153.0 μg/m^3^ in 2013) (WHO 2014). Given that billions of people worldwide are exposed to high concentrations of PM_2.5_ and that the relationship between mortality and PM_2.5_ was similar over a range of exposures in the present study and in previous studies (Burnett et al. 2014), our findings have important global public health implications. Our findings imply that important health benefits can be achieved through efforts to further reduce ambient air pollution worldwide.

We did not find strong evidence that comorbidities and medications altered the association between PM_2.5_ and mortality because the power to detect such differences was limited. Cardiovascular medications such as statins improve endothelial function, modulate inflammatory responses, maintain plaque stability, and prevent thrombus formation, all of which potentially protect against the effects of PM_2.5_ (Delfino et al. 2009; McCracken et al. 2010; Schwartz et al. 2005). Further investigation of potential interactions between cardiovascular medications and air pollution exposure in post-AMI survival is merited given the widespread use of these medications by this subpopulation.

The strengths of this study include its relatively large size and population-based representation of AMI patients in Ontario, the most populous province in Canada. In addition, we obtained extensive individual-level information including detailed clinical data and demographic and behavioral characteristics, which allowed for good control for known risk factors. Aspects of our analytic approach also reduced concerns about confounding, such as the use of spatial random-effects models. The standard Cox model yielded smaller estimates of the standard error for PM_2.5_ than those produced by the spatial random-effects model, suggesting that there was unexplained spatial variation in mortality within the cohort. By specifying nested spatial clusters to account for possible spatial dependencies among participants, the spatial random-effects models improved the estimation of the standard error for PM_2.5_ effects. In addition, our study benefited from having information on cause of death, allowing the association between PM_2.5_ and mortality to be analyzed in great detail. Furthermore, the use of satellite-based long-term average estimates of PM_2.5_ ensured virtually complete spatial coverage of PM_2.5_ exposure for the cohort.

Several limitations merit mention. First, we lacked information on individual socioeconomic status (SES) such as income and education. However, we controlled for smoking, employment status, area-level SES, and comorbidities, which may partly lie in the causal pathway between individual SES and post-AMI mortality (see Figure S1). Although we cannot rule out the possibility of residual confounding by individual SES, it is unlikely that this would substantially bias our risk estimates, and the null association with negative control outcomes did not support this possibility.

Second, the spatial pattern of PM_2.5_ was derived for the period 2001 to 2010, covering most of the follow-up period (1999–2011). We have shown previously that the spatial gradients of ambient PM_2.5_ in Ontario are stable over time and that variability in PM_2.5_ concentrations is primarily spatial rather than temporal (Chen et al. 2013). Because 78% of cohort members never moved or moved only within the city of residence, the spatial contrasts in PM_2.5_ over 2001–2010 are expected to be a reasonable representation of longer-term spatial exposures to PM_2.5_ in Ontario (Chen et al. 2013).

Third, the 10 km × 10 km resolution of the satellite-based exposure surface reduced our ability to capture the fine-scale intraurban variation in PM_2.5_ exposures that tends to occur in areas with relatively high PM_2.5_ concentrations. This low resolution may result in larger uncertainties in characterizing the concentration–response relationship at the higher end of PM_2.5_ exposures. We also did not have information on daily activity. Given the inherent imprecision of the spatially derived exposure, our assessment of exposure was likely subject to nondifferential misclassification that may have attenuated the estimates. In addition, our analyses did not consider the mixture of air pollutants to which subjects may have been exposed.

Fourth, information on most potential confounding variables was obtained at baseline only. Although we adjusted for medications at discharge and coronary revascularization during follow-up, we could not further account for post-discharge medications because the information was unavailable.

In this study, the strongest associations with PM_2.5_ appeared to be for cardiovascular-related mortality, particularly from ischemic heart disease. This finding supports that the biological pathways involved in the cardiovascular effects of PM_2.5_ (Brook et al. 2010), including systemic oxidative stress and inflammation, increased blood coagulability, enhanced thrombosis, and vascular dysfunction, may have played an important role in increasing post-AMI mortality. These responses may have a large impact on individuals who already have compromised cardiovascular systems, such as AMI patients.

## Conclusions

In summary, this study adds weight to previous observations that AMI patients are susceptible to the effects of air pollution, and it provides new evidence that the survival of AMI patients may be significantly influenced by long-term exposure to PM_2.5_, even at the relatively low levels observed in Ontario.

## Supplemental Material

(192 KB) PDF
