# Supplementary material for: Ambient Fine Particulate Matter and Mortality among Survivors of Myocardial Infarction: Population-Based Cohort Study
Source: Environ Health Perspect. 2016 May 6;124(9):1421–8. doi: 10.1289/EHP185 (PMC5010396; doi:10.1289/EHP185)
Supplement: (192 KB) PDF [file EHP185.s001.acco.pdf]

Environ Health Perspect
DOI: 10.1289/EHP185

Note to readers with disabilities: *EHP* strives to ensure that all journal content is accessible to all readers. However, some figures and Supplemental Material published in *EHP* articles may not conform to 508 standards due to the complexity of the information being presented. If you need assistance accessing journal content, please contact ehp508@niehs.nih.gov. Our staff will work with you to assess and meet your accessibility needs within 3 working days.

Supplemental Material

Ambient Fine Particulate Matter and Mortality among Survivors of Myocardial Infarction: Population-Based Cohort Study

Hong Chen, Richard T. Burnett, Ray Copes, Jeffrey C. Kwong, Paul J. Villeneuve, Mark S. Goldberg, Robert D. Brook, Aaron van Donkelaar, Michael Jerrett, Randall V. Martin, Jeffrey R. Brook, Alexander Kopp, and Jack V. Tu

Table of Contents

Burden Attributable to $PM_{2.5}$

International Classification of Diseases (ICD) Codes for Study Outcomes

Canadian Census Divisions and Census Tracts

Comparison of Spatial Resolution for Different Datasets in the Study

Additional Sensitivity Analyses

Table S1. ICD-9 and ICD-10 codes for study outcomes

Table S2. Comparison of selected characteristics between study participants who lived in the Greater Toronto Area (GTA) and those who lived outside GTA

Table S3. Comparison of models with exposure to $PM_{2.5}$ as either a linear term or a non-linear term using a natural cubic spline function, by selected causes of death

Table S4. Sensitivity analyses for the association of non-accidental mortality with every 10-$\mu g/m^3$ increase of $PM_{2.5}$

Figure S1. Directed acyclic graph showing variables of interest and their causal association

References

Burden Attributable to $PM_{2.5}$

To quantify the burden of death attributed to long-term exposure to $PM_{2.5}$, we derived attributable fraction which was applied to the number of all-natural deaths during the follow-up using the formula as follows (Lim et al. 2012; World Health Organization 2004):

$$AF = \frac{\sum_0^m P_i \times RR_i - \sum_0^m P'_i \times RR_i}{\sum_0^m P_i \times RR_i}$$

Where $\boldsymbol{AF}$ is the attributable fraction (*i.e.*, burden attributable to risk factor such as $PM_{2.5}$), $\boldsymbol{RR_i}$ is the adjusted hazard ratio at exposure level $\boldsymbol{i}$, $\boldsymbol{P_i}$ is the estimated population distribution of exposure, $\boldsymbol{P'_i}$ is the counterfactual distribution of exposure which was 4-$\mu g/m^3$, and $\boldsymbol{m}$ is the maximum exposure level.

International Classification of Diseases (ICD) Codes for Study Outcomes

In the present study, our primary outcome was non-accidental mortality, because we were interested in assessing the burden of post-AMI death attributed to air pollution. To evaluate the specificity of the association between air pollution and mortality, we also ascertained deaths from any cardiovascular disease, ischemic heart disease, and AMI, respectively. In addition, we identified deaths from accidental causes and from non-cardiopulmonary, non-lung cancer causes to serve as negative control outcomes. The *International Classification of Diseases, Ninth Revision*, ICD-9 code and *Tenth Revision*, ICD-10 code for our study outcomes are listed in Table S1.

Canadian Census Divisions and Census Tracts

Canadian census divisions are group of neighbouring municipalities joined together for the purposes of the provision of services (such as ambulance services) and regional planning. A census division corresponds to a county or a regional district.[3] In contrast, Canadian census tracts

are small and relatively homogeneous geographic units that usually comprise a population of 2,500 to 8,000 (Statistics Canada 2011). It is one of the smallest standard geographic areas for which all census data are disseminated (Statistics Canada 2011).

Comparison of Spatial Resolution for Different Datasets in the Study

Spatial resolution for different datasets used in our study is described as follows (in the order from highest to lowest):

Postal codes (a total of 269,676 in Ontario) > Census tract (2,136 in Ontario) > 10km by 10km grids in the $PM_{2.5}$ exposure surface (1,198 grids in Ontario) > Census subdivision (507 in Ontario) > Census division (50 in Ontario)

These datasets were created by different organizations for different purposes; as a result, their areas may overlap. For example, 10km by 10km grid-cells may overlap with census divisions.

Additional Sensitivity Analyses

We further investigated the possibility of adjusting for geographically-variable sociodemographic and other related health-care indicators. In doing this, we created five new sets of covariates: (1) a dichotomous indicator variable for North/South Ontario; (2) rurality; (3) neighborhood-level % of visible minority; (4) deprivation; and (5) density of family doctors. We conducted sensitivity analyses by additionally controlling for these new variables.

To classify Ontario into northern and southern regions, we created an indicator variable based on the 14 Ontario Local Health Integrated Networks (equivalent to health regions). The Local Health Integrated Networks are responsible for planning, integrating, and funding various local health care services across the province of Ontario. Of the 14 Local Health Integrated Networks,

two (North East and North West) cover the population living in northern Ontario and were combined to create the indicator (versus the rest of health regions).

To represent rurality, we created two separate variables. The first variable comprises five categories (urban core, urban fringe, rural fringe, urban area outside census metropolitan area, and rural area outside census metropolitan area) as defined by Statistics Canada. The second variable was created based on the Rurality Index for Ontario which takes into account community population density, travel time to nearest basic referral centre, and travel time to nearest advanced referral centre (Kralj 2000). The Rurality Index has a value ranging from 0 to 100, with 0-39 considered as urban and 40 and above considered rural (Kralj 2000). The Rurality Index has been used by the Ontario government and the Ontario Medical Association for determining incentive and bonus payment to physicians, and has been widely used to define urban-rural split in previous research in health system improvement.

In addition, using the 2001 Canadian Census Tract data, we derived a variable for % of visible minority. As well, we derived a variable to represent neighbourhood-level deprivation based on the Ontario Marginalization Index that has been previously developed to quantify the degree of marginalization in health and social well-being across Ontario (Matheson et al 2012). It consists of four dimensions thought to underlie the construct of marginalization: residential instability; material deprivation; dependency; and ethnic concentration. Additionally, we created a variable for density of family physicians using the Physician Database in Ontario, which contains information about all physicians in Ontario.

Furthermore, we conducted sensitivity analyses by restricting to cohort members who lived between 41.7^{o} N and 46.0^{o} N, where the vast majority of the Ontario population resides (range of

latitude in Ontario: 41.7^{o} N to 50.8^{o} N). Lastly, we conducted analyses by restricting to cohort members who lived within 5 km from any manufacturing or process facilities releasing particulate matter that meet reporting thresholds and hence are required to report to Environment Canada, according to the Canadian Environmental Protection Act (1999).

Table S1. ICD-9 and ICD-10 codes for study outcomes

Cause of death	ICD-9 code	ICD-10 code
Non-accidental	<800	A00-R99
Any cardiovascular	401-459	I10-I99
Ischemic heart	410-414	I20-I25
Acute myocardial infarction	410	I21
Non-cardiopulmonary, non-lung cancer	<401, 520-799, and not 162	A00-I09, K00-R99, and not C33-C34
Accidental	≥800	S00-Y98

Table S2. Comparison of selected characteristics between study participants who lived in the Greater Toronto Area (GTA) and those who lived outside GTA

Characteristics	Participants in GTA n=2,352	Participants outside GTA n=6,521
Mortality rate by cause of death (number of deaths per 1000 person years) *		
Non-accidental	51	59
Any cardiovascular	26	32
Ischemic heart	20	25
Acute myocardial infarction	8	10
Non-cardiovascular	25	26
Relative risk of death †		
Non-accidental (hazard ratio, 95% CI)	0.86 (0.81-0.93)	1
Any cardiovascular (hazard ratio, 95% CI)	0.80 (0.73-0.88)	1
Ischemic heart (hazard ratio, 95% CI)	0.78 (0.70-0.88)	1
Acute myocardial infarction (hazard ratio, 95% CI)	0.79 (0.66-0.94)	1
Non-cardiovascular (hazard ratio, 95% CI)	0.96 (0.86-1.05)	1
Demographic, behavioral, and comorbid characteristics		
Age (years)	66.4±13.3	67.0±12.8
Men	66	65
Smoking status		
Never smoker	29	28
Former smoker	22	24
Current smoker	35	36
Unknown	14	12
Body mass index (kg/m^2)		
<18.5	1	1
18.5-24.9	15	17
25.0-29.9	23	26
≥30.0	15	17
Unknown	46	39
Family history of coronary artery disease	30	34
Diabetes	24	25
Stroke	6	7
Characteristics of medical care		
Specialty of attending physician		
Cardiology	60	26
Internal medicine	28	32
General practice	12	42
Characteristics of hospitals		
Teaching	15	13
Community	85	78

Characteristics	Participants in GTA n=2,352	Participants outside GTA n=6,521
Small	0	9
Received coronary revascularization during follow-up	40	35
Received statins prescription at hospital discharge	37	34
Distance to nearest acute-care hospitals, mean (in meter)	4,234	7,736

Values are percent or mean ± standard deviation, unless otherwise indicated.

* Study participants lived in the Greater Toronto Area contributed to 19,721 person-years of observations whereas participants lived in all other regions of Ontario contributed to 52,380 person-years of observations. The mortality rates were presented as unadjusted rates per 1,000 person years.

† Adjusted for age and sex, with reference to participants outside Toronto, by cause of mortality.

Table S3. Comparison of models with exposure to $PM_{2.5}$ as either a linear term or a non-linear term using a natural cubic spline function, by selected causes of death

	Akaike information criterion (AIC)		
**Model**	Non-accidental	Ischemic heart	Acute myocardial infarction
Cox model with $PM_{2.5}$ as linear term [*]	31689.3	12930.4	5471.9
Cox model with **ns**($PM_{2.5}$, df=2) [†]	31691.3	12932.4	5473.8
Cox model with **ns**($PM_{2.5}$, df=3)	31692.6	12930.6	5471.0
Cox model with **ns**($PM_{2.5}$, df=4)	31692.8	12932.3	5470.1

[*] Model stratified by age and region, and adjusted for sex, marital status, employment, smoking, family history of coronary artery disease, diabetes, hyperlipidemia, hypertension, stroke, previous PCI, previous AMI, GRACE risk score, STEMI/Non-STEMI, acute pulmonary edema, length of stay, characteristics of physicians and hospitals, cardiovascular medication use, comorbidities, and area-level variables.

[†] **ns**(•) denotes natural cubic spline function and **df** denotes degrees of freedom.

Table S4. Sensitivity analyses for the association of non-accidental mortality with every 10-$\mu g/m^3$ increase of $PM_{2.5}$

Sensitivity Analysis	No. of deaths	Non-accidental mortality [*]	
		Hazard Ratio	95% CI
Adjusted for North/South indicator	4,016	1.25	1.02 - 1.54
Adjusted for urban-rural status [†]	4,016	1.22	1.02 - 1.45
Adjusted for Rurality Index for Ontario	4,016	1.22	1.02 - 1.47
Restricted to participants living between 41.7° N and 46.0° N in Ontario	3,683	1.28	1.03 - 1.59
Restricted to participants living within 5 km from facilities releasing particulate matter	3,542	1.24	1.00 - 1.54
Adjusted for deprivation index	4,016	1.24	1.03 - 1.49
Adjusted for density of family physicians [‡]	4,016	1.22	1.01 - 1.44
Adjusted for % visible minority	4,016	1.33	1.11 - 1.59

[*] A nested, spatial random-effects Cox model, stratified by age and region, and adjusted for sex, marital status, employment, smoking, family history of coronary artery disease, diabetes, hyperlipidemia, hypertension, stroke, previous PCI, AMI, GRACE risk score, STEMI/Non-STEMI, acute pulmonary edema, in-hospital care, medications, comorbidities, and area-level variables.

[†] The variable comprises five categories (urban core, urban fringe, rural fringe, urban area outside census metropolitan area, and rural area outside census metropolitan area) as defined by Statistics Canada.

[‡] At the census subdivision level (equivalent to municipalities).

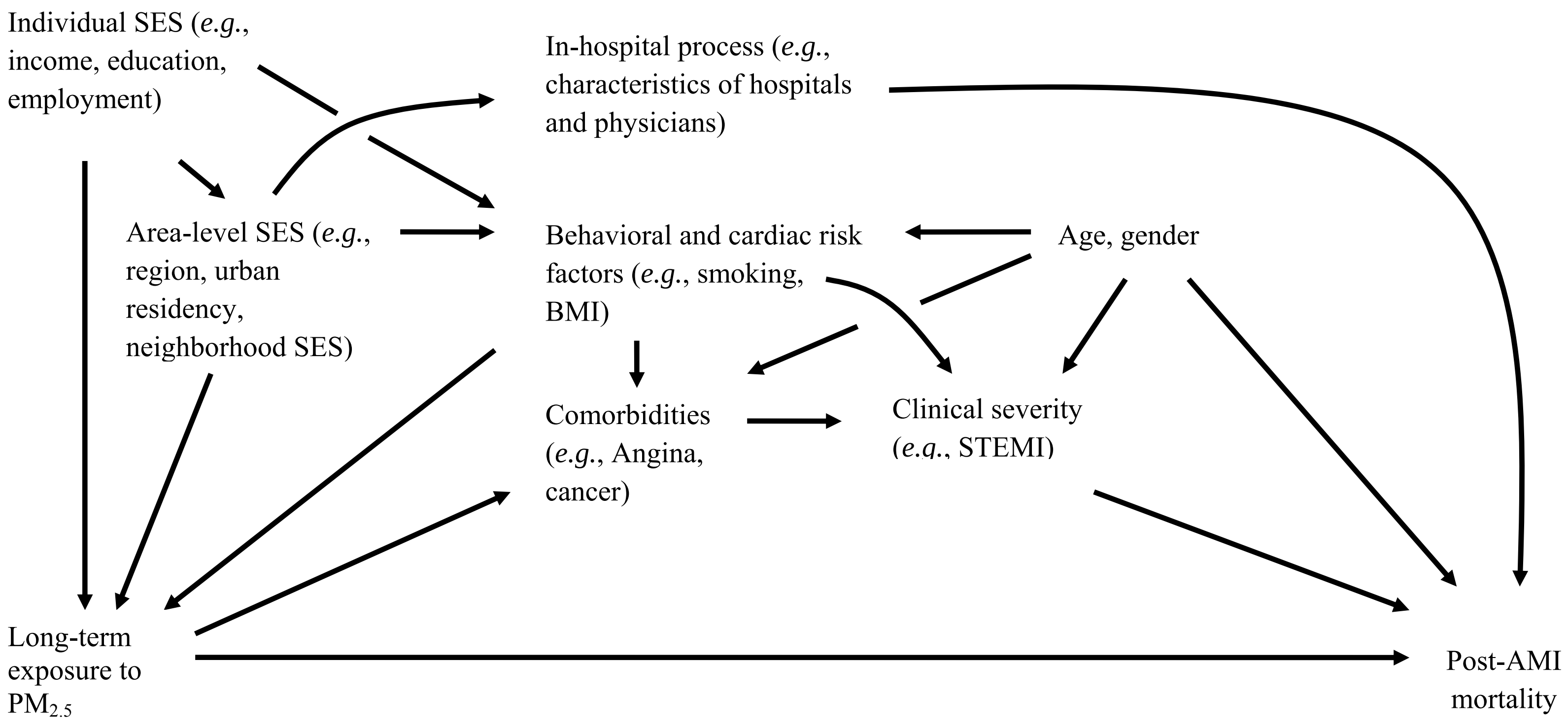

Figure S1. Directed acyclic graph showing variables of interest and their causal association

References

Kralj B: Measuring "rurality" for purposes of health-care planning: an empirical measure for Ontario. Ont Med Rev 2000;33-52.

Lim SS, Vos T, Flaxman AD, Danaei G, Shibuya K, Adair-Rohani H, et al. 2012. A comparative risk assessment of burden of disease and injury attributable to 67 risk factors and risk factor clusters in 21 regions, 1990-2010: a systematic analysis for the Global Burden of Disease Study 2010. Lancet 380:2224-2260.

Matheson FI, Dunn JR, Smith K, Moineddin R, Glazier RH. Development of the Canadian Marginalization Index: a new tool for the study of inequality. Can J Public Health 2012;103(Suppl. 2):S12-S16.

Statistics Canada. 2011. Canadian Census Dictionary. http://www12.statcan.gc.ca/census-recensement/2011/ref/dict/index-eng.cfm. [Accessed March 31, 2015].

World Health Organization. 2004. Outdoor air pollution: assessing the environmental burden of disease at national and local levels. http://www.who.int/quantifying_ehimpacts/publications/ebd5.pdf. [Accessed March 31, 2015].
